# An All‐In‐One Transient Theranostic Platform for Intelligent Management of Hemorrhage

**DOI:** 10.1002/advs.202301406

**Published:** 2023-06-04

**Authors:** Reihaneh Haghniaz, Ankit Gangrade, Hossein Montazerian, Fahimeh Zarei, Menekse Ermis, Zijie Li, Yuxuan Du, Safoora Khosravi, Natan Roberto de Barros, Kalpana Mandal, Ahmad Rashad, Fatemeh Zehtabi, Jinghang Li, Mehmet R. Dokmeci, Han‐Jun Kim, Ali Khademhosseini, Yangzhi Zhu

**Affiliations:** ^1^ Terasaki Institute for Biomedical Innovation Los Angeles CA 90064 USA; ^2^ Department of Bioengineering University of California Los Angeles CA 90095 USA; ^3^ California NanoSystems Institute University of California Los Angeles CA 90095 USA; ^4^ Mork Family Department of Chemical Engineering & Materials Science Viterbi School of Engineering University of Southern California Los Angeles CA 90007 USA; ^5^ Electrical and Computer Engineering Department University of British Columbia Vancouver BC V6T 1Z4 Canada; ^6^ College of Pharmacy Korea University Sejong 30019 Republic of Korea

**Keywords:** antibacterial, capacitive sensors, hemostatic, silk sponge, theranostic devices

## Abstract

Developing theranostic devices to detect bleeding and effectively control hemorrhage in the prehospital setting is an unmet medical need. Herein, an all‐in‐one theranostic platform is presented, which is constructed by sandwiching silk fibroin (SF) between two silver nanowire (AgNW) based conductive electrodes to non‐enzymatically diagnose local bleeding and stop the hemorrhage at the wound site. Taking advantage of the hemostatic property of natural SF, the device is composed of a shape‐memory SF sponge, facilitating blood clotting, with ≈82% reduction in hemostatic time in vitro as compared with untreated blood. Furthermore, this sandwiched platform serves as a capacitive sensor that can detect bleeding and differentiate between blood and other body fluids (i.e., serum and water) via capacitance change. In addition, the AgNW electrode endows anti‐infection efficiency against *Escherichia coli* and *Staphylococcus aureus*. Also, the device shows excellent biocompatibility and gradually biodegrades in vivo with no major local or systemic inflammatory responses. More importantly, the theranostic platform presents considerable hemostatic efficacy comparable with a commercial hemostat, Dengen, in rat liver bleeding models. The theranostic platform provides an unexplored strategy for the intelligent management of hemorrhage, with the potential to significantly improve patients' well‐being through the integration of diagnostic and therapeutic capabilities.

## Graphical abstract

A novel biocompatible and biodegradable all‐in‐one theranostic platform is developed to sense and control bleeding. The device comprises a silk fibroin (SF) sponge placed between silver nanowire (AgNW) electrodes that act as a capacitive sensor to detect bleeding and differentiate between blood and other body fluids. The device demonstrates anti‐infection properties and hemostatic efficacy comparable to a commercial hemostat.

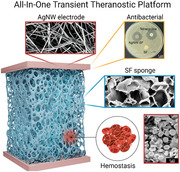

## 1 Introduction

Hemorrhage, induced by medical conditions such as traumatic injuries, post‐surgical concerns, stroke, and gastrointestinal wounds, can cause various complications, including hypothermia, coagulopathy, and organ failure, and even leads to death if not treated early.^[^ ^1^ ^]^ The body's natural clotting cascade can stop minor bleeding.^[^ ^2^ ^]^ However, for severe hemorrhage, timely detection and effective control of blood loss with hemostatic agents could be lifesaving and reduce the mortality rate of the patients.^[^ ^2^, ^3^ ^]^ An ideal hemostat should offer rapid hemostasis, early detection of bleeding, excellent anti‐infection performance, minimal inflammation, active coagulation management, and good biocompatibility.^[^ ^4^ ^]^

To date, a wide variety of hemostatic materials have been investigated, including cotton, gauze, bandage, powder, and tourniquets. However, most have limited hemostatic efficiency and poor hemorrhage control for deep, irregularly shaped, and non‐compressible wounds.^[^ ^5^ ^]^ A new generation of sponge‐like hemostatic materials with shape‐memory properties has emerged for effective hemorrhage control. The porous structure of such hemostatic sponges allows the absorption of a large quantity of blood while maintaining their original dimension and structures. Moreover, the highly compressible sponges apply pressure on blood vessels and facilitate blood coagulation, reducing the incidence of postoperative issues.^[^ ^6^ ^]^ Xstat is a representative example of a commercially available cellulose‐based shape‐memory hemostatic sponge that can be injected into a deep wound, expands its volume, and stops bleeding. However, Xstat sponges are non‐degradable, and they must be removed from the body by surgery, resulting in potential secondary damage and additional pain to the patient.^[^ ^7^ ^]^ Dengen, Spongostan, Gelfoam, and DSI sponges are commercialized gelatin‐based hemostatic sponges that are biodegradable but suffer from thrombogenic complications, allergic reactions, and inflammatory responses.^[^ ^4b^ ^]^

Therefore, exploring other sources of biodegradable and biocompatible hemostatic materials for fabricating shape‐memory sponges has an unmet clinical need. Silk fibroin (SF) is a natural protein derived from *Bombyx mori* (Silkworm).^[^ ^8^ ^]^ SF exhibits excellent mechanical properties and minimal inflammatory reactions.^[^ ^9^ ^]^ The ability of SF to enhance cell adhesion, proliferation, differentiation, and migration has led them to be increasingly used in wound dressings and wound healing agents.^[^ ^4^, ^9^, ^10^ ^]^ Moreover, the hemostatic activity of SF in binding with fibrinogen and blood platelets triggering clotting cascade has been reported in several studies.^[^ ^11^ ^]^ SF can be made in sponge form, which offers a high‐volume micro‐porous structure beneficial for hemostatic application by trapping red blood cells (RBCs) and blood sorption.^[^ ^12^ ^]^ Furthermore, SF is biodegradable due to its polypeptide chain, and its degradation rate can be tuned based on the SF concentration and degree of crystallization,^[^ ^13^ ^]^ which make it a desirable platform for sensor integration. One can adjust the degradation rate at which SF‐based hemostats remain in the body for a longer period, enabling extended monitoring of hemorrhage. In addition, SF contains various functional groups,^[^ ^10a^ ^]^ making it easy to modify its surface,empower it to detect bleeding and provide extra antibacterial properties to prevent post‐surgery infection.

Inflammation caused by microbial infection after uncontrolled hemorrhage is a considerable challenge. Therefore, developing hemostatic agents that efficiently prevent infection has clinical application value.^[^ ^14^ ^]^ Silver materials in the nanoscale have great antibacterial properties, mainly by releasing silver ions (Ag^+^) from their surfaces to combine with the negatively charged bacteria membranes to interrupt normal bacteria functions or catalyze the production of reactive oxygen species to interfere with enzymes and DNA functions.^[^ ^15^ ^]^ We have previously synthesized AgNWs offering antibacterial properties.^[^ ^16^ ^]^ Integrating AgNW and SF can provide synergistic antibacterial and hemostatic properties to the device. Moreover, AgNW is highly conductive and mechanically flexible, making them ideal for flexible electronics applications.^[^ ^16^, ^17^ ^]^

Although hemostatic materials can reduce mortality by coagulating the blood at the injury site, they lack the capabilities to alert the hemorrhage after therapy. Active monitoring and detecting bleeding after the surgery are essential for patients' health outside the hospital.^[^ ^18^ ^]^ To our knowledge, no available hemostatic device can provide concurrent hemorrhage sensing and blood coagulation. Herein, we report an all‐in‐one theranostic device composed of an AgNW functionalized shape‐memory SF sponge, which can simultaneously offer diagnosis and control of bleeding and its consequence infection.

## 2 Results and Discussion

### 2.1 Structure Design, Composition Characterization, and Morphology Control of AgNW‐SF Theranostic Device

**Figure 1:**
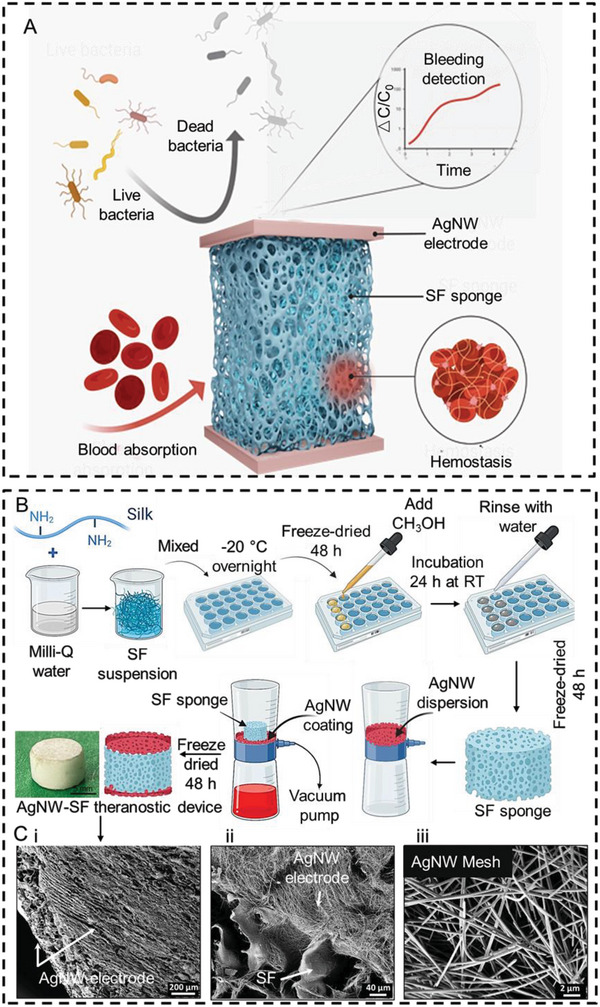
Structural design, fabrication workflow, and composition characterizations of AgNW‐SF theranostic device. A) Conceptual illustration of the AgNW‐SF theranostic device for intelligent hemorrhage management. B) The fabrication process of the AgNW‐SF theranostic device. C, i) Top view SEM image of the AgNW‐SF theranostic device. ii) The magnified cross‐sectional SEM image reveals a thin AgNW electrode on the surface of the porous SF sponge, as indicated with arrows. iii) Magnified SEM image of AgNWs to confirm their uniformity in diameter and length.

As conceptually displayed in **Figure** **1**A, the all‐in‐one AgNW‐SF theranostic device integrates hemostasis, antibacterial activity, and bleeding detection abilities for the management of the hemorrhaging wound. The device consists of three functional components, including the top and bottom AgNW layers for conducting electrodes and antibacterial agents, and the middle silk sponge for efficient hemostasis. The device also serves as a sandwiched capacitive sensor for hemorrhage detection. The fabrication procedure of the AgNW‐SF theranostic device is illustrated in Figure 1B, which is further detailed and summarized in the Experimental Section. To prepare different concentrations of SF dispersions (3%, 4%, and 5% w/v), commercially available SF precursor was diluted with Milli‐Q water and then distributed in 48‐well plates, followed by overnight freezing at −20 °C. The frozen samples were freeze‐dried for 48 h and subsequently crystallized with methanol for 24 h at room temperature (RT). The crystallization significantly affects the mechanical strength and degradation rate of the SF sponges. A longer time of methanol treatment induces a larger amount of *β*‐sheets in the SF matrix, resulting in an enhancement of mechanical strength and relatively slow degradation.^[^ ^19^ ^]^ By contrast, non‐crystalized freeze‐dried SF sponges dissolve or shrink quickly in wet physiological conditions.^[^ ^20^ ^]^ After the crystallization step, the methanol residues were rinsed with Milli‐Q water, and the samples were further freeze‐dried for 48 h to form porous structured SF sponges. To assemble the AgNW‐SF‐based capacitive sensor, AgNWs were diluted using ethanol and sonicated for 10 min to form a homogenous suspension. A thin conductive film was constructed by filtering the AgNWs from the suspension onto a porous membrane via vacuum filtration. The AgNW electrode was then transferred to the SF sponge by applying pressure to the back side of the membrane and forcing intimate contact with the SF sponge. We used the same procedure to deposit the AgNW thin film on the opposite side of the SF sponge. The coating procedure was repeated three times on each side of the SF sponges to ensure their sufficient conductivity. The surface morphology was characterized by scanning electron microscopy (SEM). Figure 1C(i,ii) shows the top and magnified cross‐sectional view of the AgNW‐SF theranostic device, displaying a porous structure of freeze‐dried SF sponge and a thin layer of AgNW electrode, which is ≈10–15 µm thick and located on the sponge's surface. The magnified SEM image in Figure 1C(iii) clearly displays the filament structure of the AgNW mesh with a diameter of ≈113 nm and length of ≈24 µm, which are in agreement with our previous publication.^[^ ^16a^ ^]^ Figure S1 (Supporting Information) shows Fourier‐transform infrared spectroscopy spectra of the freeze‐dried SF sponges before and after the deposition of AgNW electrodes. The pure SF sponges demonstrated strong transmittance bands in the amide I region (1600–1700 cm^−1^), associated with the vibrations of the amide bonds in the protein's secondary structure. A strong peak in the amide II region (1500–1600 cm^−1^) was also observed, which is associated with the bending vibrations of amide bonds.^[^ ^21^ ^]^ It also shows a reduction in the intensities of amide I and amide II bands after the deposition of AgNW electrodes on the SF sponge surfaces, which can be attributed to the interaction of the Ag^+^ ions with C=O, COO^−^, and NH groups of the protein.^[^ ^22^ ^]^ More importantly, the AgNW‐SF theranostic device has excellent manufacturability that can be tailored to different structures by direct laser engraving (Figure S2, Supporting Information), empowering their applications in developing personalized devices.

### 2.2 Mechanical and Shape‐Memory Characteristics of AgNW‐SF Theranostic Devices

**Figure 2:**
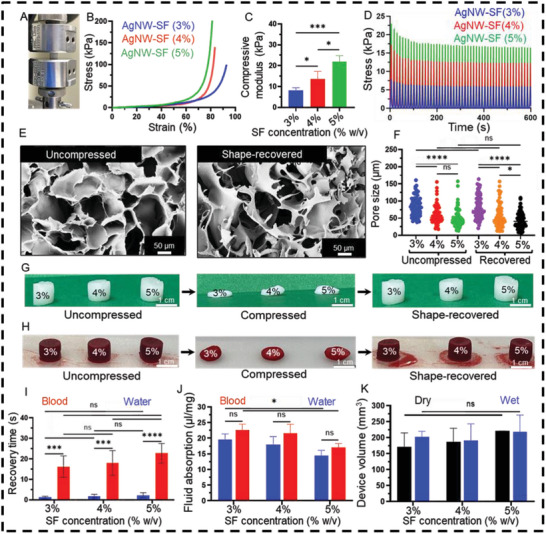
Mechanical and shape‐memory properties of AgNW‐SF theranostic devices. A) A digital image of the compression test setup. B) Representative compressive stress‐strain curves of the devices with SF concentrations varied from 3% to 5% w/v. C) The compressive modulus of AgNW‐SF theranostic devices fabricated with varying SF concentrations. D) Cyclic compression test of AgNW‐SF theranostic devices after 50 compression cycles. E) SEM images representing pore structures of the AgNW‐SF theranostic device (with 4% SF) before compression and after shape‐recovery. F) Pore size distribution of as‐synthesized uncompressed AgNW‐SF devices versus water‐absorbed recovered AgNW‐SF theranostic devices. G) Photographs of the water‐triggered shape‐recovered AgNW‐SF devices at different concentrations of SF. H) Photographs of the blood‐triggered shape‐recovered AgNW‐SF after compressing. I) Recovery time of the AgNW‐SF devices after absorption of water or blood. J) Water and blood absorption capability of AgNW‐SF devices fabricated with varying concentrations of SF. K) The total volume of AgNW‐SF devices before and after water absorption, labeled as dry and wet, respectively. When appropriate, the significant differences were analyzed for *n* ≥ 3, using one‐way or two‐way ANOVA with multiple comparisons test. Asterisks indicate statistically significant results with *p*‐values < 0.05 (*), <0.01 (**), <0.001 (***), or <0.0001 (****). The “ns” indicates no significant differences.

A good hemostatic sponge should have desirable mechanical strength in order to preserve their original shapes under compression applied by the wounded tissue.^[^ ^3^ ^]^ To examine the stability and reliability of the AgNW‐SF theranostic devices under physiological load‐bearing conditions, hydrated devices were subjected to the uniaxial compressive test, as shown in **Figure** **2**A. We investigated the effect of SF concentrations (3%, 4%, and 5% w/v) on the compressive strength of the AgNW‐SF theranostic devices. The representative stress–strain curves in Figure 2B demonstrate the sponge‐like behavior of all the devices. Namely, the stress showed an elastic trend followed by an exponential increase due to the buckling and densification of pore walls. By increasing the SF concentration from 3% to 5% w/v, the compressive modulus was significantly increased from 8.1 kPa to 22.0 kPa, i.e., a 2.5‐fold improvement in compressive modulus (Figure 2C). A cyclic compressive loading at the strain amplitude of up to 50% was performed under hydrated conditions (Figure S3A–C, Supporting Information). The results suggested less hysteresis with a decrease in SF concentration. Variation of stress with time was plotted for samples with different SF concentrations in Figure 2D. A more significant stress relaxation was associated with high SF concentrations (i.e., 5% w/v) due to the larger irreversible deformations in denser polymeric networks. The shape‐recovery performance primarily arises from the highly reversible porous structure change.^[^ ^5a^ ^]^ Therefore, the porous structure of the as‐synthesized uncompressed AgNW‐SF theranostic devices and the devices recovered after 50 cycles of the compressive stress are shown in Figure 2E and Figure S4 (Supporting Information). Figure 2F exhibits no apparent difference in the pore size distribution of the uncompressed and recovered AgNW‐SF theranostic devices. The average pore size of the as‐synthesized AgNW‐SF devices estimated from SEM images is ≈100 µm. The pore size was significantly decreased by increasing the SF concentration from 3% to 5% w/v. Furthermore, the pore size of the recovered AgNW‐SF devices was not obviously affected by the compressive load, suggesting the excellent elasticity of the device.

To further examine the shape‐memory feature of the device, water‐ and blood‐initiated structure recovery behaviors of the hydrated AgNW‐SF devices were assessed and summarized in Figure 2G, H. The compressed AgNW‐SF devices could retain their shape‐fixed structures after releasing the external force, and they could recover their original shape with an excellent recovery ratio upon the absorption of water or blood (Movie S1 and Figure S5A, Supporting Information). As shown in Figure 2I, upon water absorption, the devices were recovered quickly within a few seconds, regardless of the SF concentration. However, upon absorption of blood, the recovery times were significantly prolonged to tens of seconds due to the higher blood viscosity, as demonstrated by other reports.^[^ ^3^, ^23^ ^]^ We further freeze‐dried the compressed AgNW‐SF devices and investigated their shape‐recovery behavior. As shown in Figure S5B (Supporting Information), the devices could recover their original shape upon blood absorption, comparable to the hydrated compressed devices.

Furthermore, the water and blood absorption capability of the devices is shown in Figure 2J. It can be clearly seen that the absorbability of the devices was independent of the fluid type, meaning that the devices could absorb equal volumes of either water or blood at each concentration. However, by increasing the concentration of SF from 3% w/v to 5% w/v, the device significantly diminishes its fluid absorption capability, which could be attributed to the markedly smaller pore size and stiffer structure at the concentration of 5% w/v, as discussed in Figure 2C,F. As a result, all the devices could rapidly absorb water by ≈100–140 times of their original dry weight within the first 3 min (Figure S5C, Supporting Information). The AgNW‐SF devices with 3% and 4% w/v SF absorbed more water than the AgNW‐SF device with 5% w/v SF. More importantly, the device volume did not significantly change upon water absorption, and no dimensional change was observed between the dry and wet devices, suggesting non‐swelling behavior (Figure 2K).

These results suggest that the AgNW‐SF device has excellent shape‐memory and blood uptake capability, making it desirable to rapidly absorb blood and maintain pressure on the wound to promote effective hemostasis. Furthermore, the minimal dimensional changes of the theranostic devices after fluid absorption is an advantageous feature for capacitance sensing. The fact that the blood absorbability could be governed by the concentration of SF in the AgNW‐SF device, the concentration of AgNW‐SF (3%) and AgNW‐SF (4%) w/v are more promising than AgNW‐SF (5%) for their use in the hemostatic application. However, in this study, we used AgNW‐SF (4%) devices for in vivo application since they possess a higher compressive modulus than AgNW‐SF (3%) devices, matching better with Young's modulus of the liver tissue.^[^ ^24^ ^]^

### 2.3 In Vitro Anti‐Infective Efficacy of AgNW‐SF Theranostic Devices

**Figure 3:**
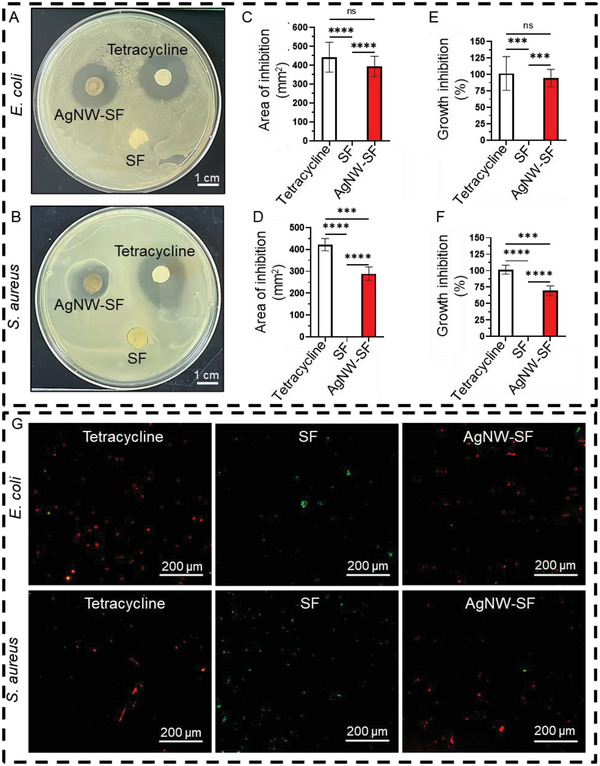
Antibacterial efficacy of AgNW‐SF (4%) theranostic devices. A) Photograph shows the zone of inhibition after challenging *E. coli*, and B) *S*. *aureus* for 24 h with the AgNW‐SF theranostic devices. Tetracycline, a broad‐spectrum antibiotic, was used as a positive control, and SF without an AgNW electrode was a negative control. C) Area of the bacterial zone of inhibition after subjecting *E. coli*, and D) *S. aureus* with the AgNW‐SF devices for 24 h. E) *E. coli* growth inhibition after subjecting to the AgNW‐SF device for 24 h. F) *S. aureus* growth inhibition after subjecting to the AgNW‐SF device for 24 h. G) Live/dead fluorescent images of bacteria after challenging with tetracycline, SF negative control, and AgNW‐SF device for 6 h. Live bacteria are stained with SYTO 9 (green), and dead bacteria are stained with propidium iodide (red). Data are displayed as mean values of 3 replicates ± standard deviation. The significant differences were analyzed by one‐way ANOVA followed by Tukey's multiple comparisons tests. Asterisks indicate statistically significant results with *p*‐values <0.05 (*), <0.01 (**), <0.001 (***), or <0.0001 (****). The “ns” indicates no significant differences.

AgNW is reported as a broad‐spectrum anti‐infective material in the literature.^[^ ^15^, ^16^, ^25^ ^]^ To evaluate the anti‐infective property of the AgNW‐SF theranostic devices, the two most prevalent sources of bacterial infection after surgery, *Staphylococcus aureus* (*S. aureus*, Gram‐positive) and *Escherichia coli* (*E. coli*, Gram‐negative),^[^ ^26^ ^]^ were subjected to the hydrated device (with SF concentration of 4% w/v). Before the bacterial culture, all the AgNW‐SF devices were sterilized under UV light for ≈1 h and soaked in sterile Milli‐Q water for one week to allow for the possible release of Ag^+^ ions. Tetracycline (60 µg mL^−1^) was used as a positive control, and SF sponge (4% w/v) served as vehicle/negative control. In the zone of inhibition test, an anti‐infective effect was detected by a clear bacteria‐free zone surrounding the AgNW‐SF devices comparable with tetracycline (**Figure** **3**A,B). As shown in Figure 3C, the area of inhibition in *E. coli* culture was not significantly different between tetracycline (442.18 mm^2^) and AgNW‐SF devices (392.96 mm^2^). However, in the *S. aureus* culture, tetracycline showed a significantly larger area of inhibition (421.82 mm^2^) compared to the AgNW‐SF devices (289.09 mm^2^), as evident in Figure 3D. Overall, the AgNW‐SF theranostic devices demonstrated ≈95% antibacterial effect against *E. coli* (Figure 3E) and ≈70% antibacterial activity against *S. aureus* (Figure 3F). In agreement with our findings, Kim et al. also reported stronger antibacterial efficacy of electron beam irradiated AgNW against *E. coli* than *S. aureus*.^[^ ^15a^ ^]^ The possible reason for the observed stronger anti‐infective effect of AgNW against *E. coli* could be attributed to the different cell wall structure of the Gram‐negative (*E. coli*) and Gram‐positive (*S. aureus*) bacteria, allowing different interactions between the AgNW and the cell wall.^[^ ^27^ ^]^ Although the exact anti‐infective mechanism of AgNW is not known, the release of the Ag^+^ ions from the oxidized Ag might be a possible reason.^[^ ^15a^ ^]^

Further investigation of the anti‐infective activity of AgNW‐SF devices by live/dead assay indicated a large number of dead bacteria (stained red) after culturing *E. coli* or *S. aureus* on the surface of AgNW electrodes (Figure 3G), which were comparable with the bacteria‐killing effect of broad‐spectrum antibiotic, tetracycline. However, the SF sponges showed a maximum number of live bacteria that stained green. These results are aligned with our previous findings in which we showed fair anti‐infective efficacy of the AgNW electrodes in vitro and in vivo using an infected wound mice model.^[^ ^16a^ ^]^

### 2.4 Assessment of Hemocompatibility, Cytocompatibility, Biodegradation, and Systemic Toxicity of AgNW‐SF Theranostic Devices

**Figure 4:**
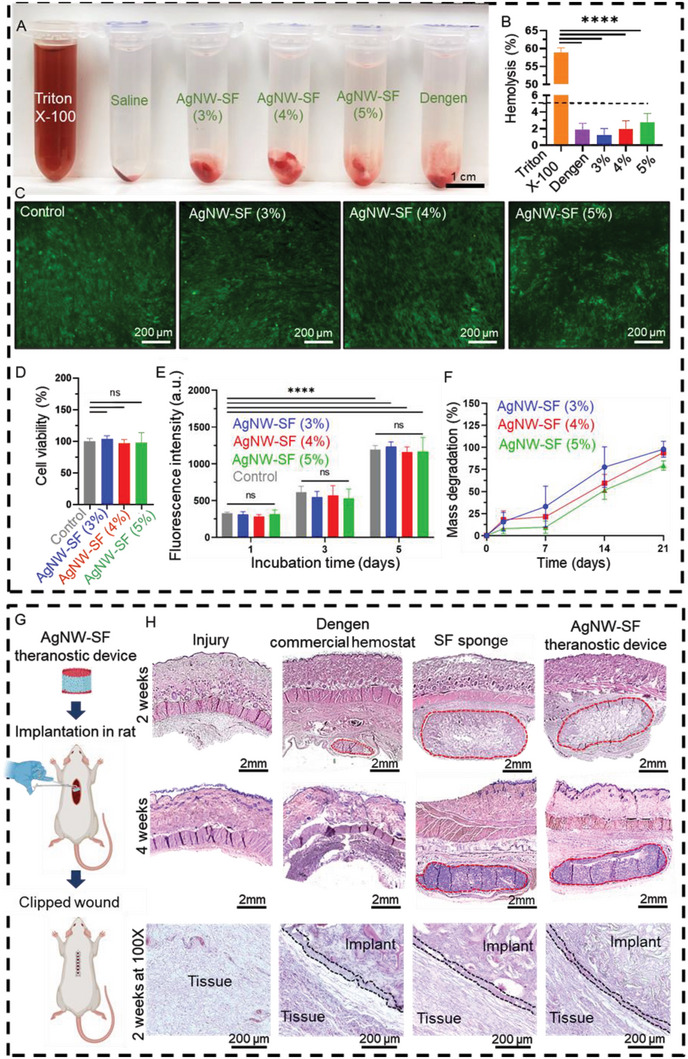
Hemocompatibility, cytocompatibility, and biodegradation of AgNW‐SF theranostic devices. A) An in vitro hemolysis assay using diluted human blood exposed to AgNW‐SF devices and Dengen, versus Triton‐X‐100 and saline as a positive and negative control, respectively. B) Quantified hemolysis for AgNW‐SF devices. The accepted limit of hemolysis (5%) is shown by a dashed line. C) Live/dead fluorescent images of human dermal fibroblast cells (HDF) cultured for 5 days on AgNW‐SF devices versus control cells (on tissue culture plates). D) Quantified HDF cell viability on day 5 of incubation with AgNW‐SF devices. E) Metabolic activity of the HDF cells was estimated using PrestoBlue assay after 1, 3, and 5 days incubation with varying concentrations of AgNW‐SF. F) In vitro mass degradation of the devices in DPBS containing 2 U mL^−1^ protease. G) A schematic illustrating subcutaneous implantation of AgNW‐SF device in a rat model. H) Histology images of the commercial hemostat, SF sponge, and AgNW‐SF device stained with H&E after 2 and 4 weeks of implantation in rats. Red dashed lines show the boundaries of the implants with tissue. The black dashed lines in the 100× magnified images show fibrous capsules. Data are displayed as mean values of 4 replicates ± standard deviation. The significant differences were analyzed by one‐way or two‐way ANOVA with multiple comparisons test, when appropriate. Asterisks indicate statistically significant results with *p*‐values <0.05 (*), <0.01 (**), <0.001 (***), or <0.0001 (****). The “ns” indicates no significant differences.

In the process of designing hemostats, evaluating hemocompatibility is crucial for determining their potential toxicity to RBCs.^[^ ^28^ ^]^ Therefore, a standard hemolysis test was performed by exposing AgNW‐SF theranostic devices to the diluted sodium citrate‐treated whole human blood for 2 h at physiological temperature. The toxic effect was visually investigated by the appearance of red‐colored supernatant after centrifugation due to the released hemoglobin from the ruptured RBCs. As can be clearly seen in **Figure** **4**A, no significant color change was observed in the supernatant after incubating the AgNW‐SF devices with blood, and RBCs were effectively sedimented through centrifugation, similar to the saline negative control, and Dengen, a commercial hemostat. However, the positive control, Triton X‐100, showed reddish supernatant, attesting to its hemolytic effect. RBCs' disruption was further evaluated with colorimetric analysis, and the calculated percent hemolysis induced by AgNW‐SF devices is plotted in Figure 4B. Despite the observed concentration‐dependent hemolytic effects of SF, the hemolysis level of the AgNW‐SF devices at all the tested concentrations was below the acceptable hemolysis limit (i.e., ≤ 5%),^[^ ^29^ ^]^ similar to the Dengen, commercial hemostat. These findings suggest that AgNW‐SF devices can be further investigated for their hemostatic ability without disrupting RBCs.

Another key factor in designing hemostatic devices is biocompatibility.^[^ ^30^ ^]^ Therefore, we evaluated the biocompatibility of AgNW‐SF devices both in vitro and in vivo to ensure that no toxicity was introduced to the cells or tissues by traces of the SF or leaching of the AgNW. For the in vitro cytocompatibility test, human dermal fibroblasts (HDF) were cultured in the porous structures of the pure SF sponges or AgNW‐SF devices at 37 °C for up to 5 days. Figure 4C shows fluorescently labeled live/dead images of the cells with prominent well‐spread live cells in green and minimal round‐shaped dead cells in red, indicating great cytocompatibility of the AgNW‐SF devices. The cell viability by day 5 was found to be >90%, with non‐significant differences between varying concentrations of the device and the control (i.e., cells seeded in the tissue culture plate), as shown in Figure 4D and Figure S6A. Metabolic activity of the cells measured by PrestoBlue assay indicated a consistent rise in fluorescence intensity from day 1 to day 5 of the culture, suggesting effective proliferation of the cells (Figure 4E, and Figure S6B, Supporting Information). The AgNW electrodes did not affect the cytocompatibility of the pure SF.

The implanted medical devices for hemorrhage management should also be biodegradable and allow the hemorrhaging wound to regenerate and heal naturally.^[^ ^3^, ^5^ ^]^ However, for the possible diagnostic application of bleeding after surgery, the device should not be degraded quickly, and the degradation rate should be ideally matched with the time span of the potential bleeding risk of the injured tissue, which is more prevalent between 24 h to 12 days after abdominal surgery.^[^ ^31^ ^]^ Since the crystalized SF sponges are well known for their slow degradation,^[^ ^19^, ^32^ ^]^ we initially assessed the in vitro degradation rate of our AgNW‐SF devices in Dulbecco's phosphate‐buffered saline (DPBS) containing 2 U mL^−1^ protease enzyme before testing them in vivo. Figure 4F presents the time‐ and concentration‐dependent degradation rate of the AgNW‐SF devices. The AgNW‐SF devices containing 3% and 4% w/v of SF underwent faster degradation and lost ≈94% of their initial mass in three weeks, whereas AgNW‐SF (5%) demonstrated slower degradation by 75% in the same period.

Further, in vivo biodegradation and biocompatibility were evaluated by implanting AgNW‐SF (at SF concentration of 4% w/v) devices in the subcutaneous pockets created in the dorsal skin of the rats, followed by external clipping to close the incision, as schematically shown in Figure 4G. Hematoxylin and eosin (H&E) staining of the retrieved devices and the surrounding tissues after 2 weeks of implantation revealed prolonged degradation of the pure SF sponge and AgNW‐SF theranostic device as compared with a gelatin‐based commercial hemostatic sponge, Dengen (Figure 4H, 2 weeks). The border between the materials and tissue (marked by red dashed lines in Figure 4H) shows approximately complete degradation of Dengen by week 4, while both the SF sponge and AgNW‐SF device held their integrity (Figure 4H, 4 weeks). In the 100X magnified images of 2 weeks post‐implantation, mild to intermediate inflammatory responses were found surrounding both SF‐based implants as well as Dengen commercial hemostat. Several layers of macrophages were observed, mostly restricted to the area surrounding implants. There were also cells infiltrating into the implanted samples due to the spongy, porous structure of the materials, facilitating cell infiltration and early stages of tissue integration. No multinucleated foreign body giant cells were observed in the sections neither around the SF‐based implants nor Dengen. A thin connective tissue layer was observed surrounding all the implanted samples showing the early stages of the fibrotic capsule (indicated by black dashed lines in Figure 4H). After 4 weeks of implantation, mild inflammatory responses were still present. However, proliferation and remodeling were more prominent by extensive cell infiltration into the SF‐based implants and reduction in thickness of the fibrous connective tissue. Compared to the gelatin‐based commercial hemostat, the slow degradation of the SF‐based implants signifies their potential for prolonged sensing application. In addition, slow degradation can reduce the risk of adverse effects associated with the sudden release of byproducts.

To determine the nature of inflammatory cells infiltrating the fibrous capsule surrounding AgNW‐SF devices, we conducted immunofluorescence staining to analyze T‐cell and macrophage infiltration (Figure S7, Supporting Information). Our findings revealed that CD3^+^ T‐cells and CD68^+^ macrophages were scattered throughout the experimental samples, with both cell types predominantly observed at week 2 and gradually diminished by week 4 with no significant difference from the injury control. To figure out if the AgNW‐SF devices serve as a substrate for the production of new extracellular matrix due to the presence of active fibroblasts, Masson's Trichrome staining was performed 2 weeks and 4 weeks after implantation. The histological study revealed a small amount of collagen deposition (blue stain) inside the sponge at 2 weeks post‐implantation (Figure S8A, Supporting Information). However, after 4 weeks, a large amount of collagen was clearly visible within sponge cavities (Figure S8B, Supporting Information). The presence of active fibroblasts was consistent with the extensive collagen deposits at 4 weeks observed by H&E staining and distinct from the dense, cable‐like collagen organization typically found in scar tissues.

To further investigate adverse effects or toxicity of the subcutaneously implanted AgNW‐SF devices, the animals were monitored for up to 4 weeks, and the main organs, including liver, lungs, heart, kidney, and spleen, were harvested and stained with H&E, 2 weeks and 4 weeks after device implantation. No behavioral change, allergic responses, weight loss, or death were observed in any of the animals. We also did not find any major signs of inflammation or abnormalities in the histological sections of the harvested tissues, as shown in Figure S9 (Supporting Information).

It is worth noting that subsequent to the degradation of AgNW‐SF devices, renal elimination may be a probable excretion pathway for AgNW, as reported for other nano‐silvers. Additionally, the liver's uptake of AgNW and its subsequent excretion in bile may represent another potential excretion route.^[^ ^33^ ^]^ Nevertheless, further in‐depth and long‐term studies are required to investigate the fate of AgNWs in vivo and elucidate their biodistribution over time.

### 2.5 In Vitro Hemorrhage Detection with AgNW‐SF Theranostic Devices

The AgNW‐SF theranostic device can also serve as a capacitive sensor, empowering detection and continuous monitoring of potential hemorrhage at the injury/wound site after surgery. To construct such sandwich structured capacitive sensor, AgNW electrodes coated on the top and the bottom sides of the SF sponge act as conductive parallel plates while the middle SF sponge works as an insulator. The AgNW electrodes exhibited high electrical conductivity with a sheet resistance of approximately 5 Ω/sq. Furthermore, their conductivity remained stable even after undergoing multiple hydration and freeze‐drying cycles, suggesting the excellent durability of the final AgNW‐SF theranostic devices (Figure S10, Supporting Information).

**Figure 5:**
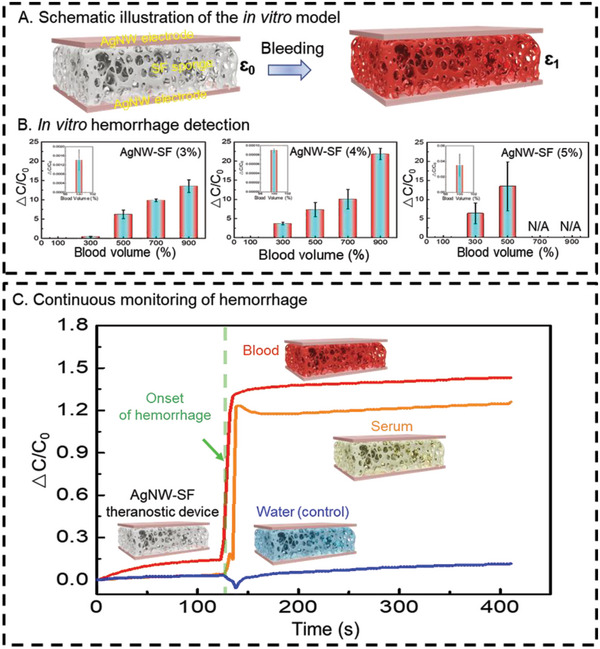
In vitro monitoring of the hemorrhage with AgNW‐SF theranostic device. A) A capacitance sensor is created by mounting two AgNW electrodes on the top and bottom surfaces of the SF sponge. The device detects bleeding when blood penetrates the SF sponge and modifies the dielectric constant from *ε*_0_ to the higher value of *ε*_1_. B) Bleeding detection potential of different concentrations of AgNW‐SF devices. C) Continuous monitoring of hemorrhage by distinct variation in capacitance after absorption of blood versus serum and water.

Capacitance is termed as C = *ε* A/d, where *ε* is the dielectric constant of the SF sponge between the AgNW electrodes, A is the surface area of the AgNW electrodes, and d is the distance between the electrodes. The bleeding can be rapidly detected by an obvious capacitance change. During bleeding, the blood would penetrate into the SF sponge, significantly increasing its initial dielectric constant *ε*, causing a correlated capacitance change in response to the bleeding, as illustrated in **Figure** **5**A. The capacitance of the AgNW‐SF device (at 4% SF) consistently increased with frequent absorption of an equal volume of blood and continued to rise until the capacitive sensors met their maximal detection limit. A similar trend was observed for AgNW‐SF devices (3% and 5%). However, the AgNW‐SF device (5%) saturated much faster, with only 500% of blood volume, indicating its narrow working range. However, the AgNW‐SF (3%) device can detect blood as much as the AgNW‐SF (4%) device, the AgNW‐SF (4%) device has a higher sensitivity with a drastic response in capacitance change during the bleeding and lower detection range (100% blood volume), which might be attributed to its more desired tissue‐like mechanics and porous structures (Figure 5B). Note that some errors may happen because of the inconsistency of blood penetration into the device.

Although the AgNW‐SF theranostic devices hold massive promise in the early detection of hemorrhage, other bodily fluids may also have access to the device during use, which may introduce noises to the signals. To address this concern, we further investigated how sensing performance is affected by the presence of other bodily fluids, including serum and water. To this end, an equal volume (100 µL) of different fluids was injected into the hydrated AgNW‐SF device (4%). Figure 5C shows a distinction in capacitance change between blood and serum or water that could help identify the possible interruptions made by other fluids in the final collected signal, enabling the accurate detection of the onset of the hemorrhage. To assess the efficacy of the AgNW‐SF devices in detecting hemorrhage subsequent to saturation with other bodily fluids, we conducted an experiment where the freeze‐dried devices were immersed in serum. As plotted in the graphs, the obtained capacitance values were subtracted from the initial capacitance (*C*_0_) to calculate ∆*C*/*C*_0_. Following saturation, the devices were transferred to a pool of whole blood while the capacitance was continuously monitored in real‐time. Notably, a substantial increase in capacitance was observed upon transitioning from serum to blood, indicating the onset of hemorrhage. (Figure S11, Supporting Information). The higher capacitance values obtained for whole blood as compared with the serum could be attributed to the richer source of electrolytes in the whole blood, such as fibrin, clotting factors, and hemoglobin, which are not present in the serum.^[^ ^34^ ^]^ Besides, we have to clarify that this work offers a proof‐of‐concept result that shows the capabilities of the non‐enzymatic, real‐time monitoring of the hemorrhage with the AgNW‐SF theranostic device. A more systematic study will still need to be done, including introducing more advanced machine learning algorithms to improve data accuracy and integrating a miniaturized wireless readout to meet excellent practicability.

### 2.6 In Vitro and In Vivo Hemostatic Efficacy of AgNW‐SF Devices

**Figure 6:**
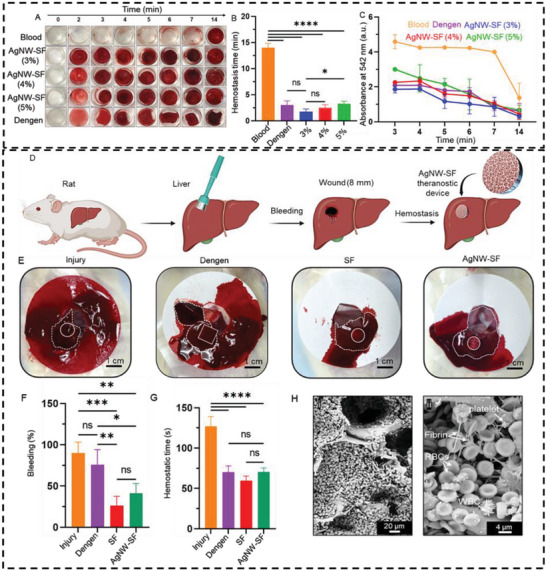
Hemostatic efficacy of AgNW‐SF theranostic devices. A) Comparison of blood clotting time in different experimental groups. B) Quantification of in vitro hemostatic time. C) Clot formation analysis by reading the absorbance of the diluted supernatant collected after washing the blood clot. D) Application of AgNW‐SF devices to stop hemorrhage in rat liver bleeding model. E) Photographs of the blood absorbed by the filter papers after treating the hemorrhaging wound with different experimental hemostats. F) The amount of bleeding after treatment of the hemorrhaging wound with SF‐based hemostats as compared with the untreated injury group and commercial hemostat, Dengen. G) Hemostatic time, corresponding to the time required to stop bleeding of the injured liver by different experimental materials. H, i,ii) SEM images represent the blood clot features in the AgNW‐SF device. Fibrin, platelets, red blood cells (RBCs), and white blood cells (WBCs) are shown by arrows in the magnified image.

Most commercially available hemostats for clinical applications are not ready to use and rely on in situ crosslinking mechanisms or do not have sufficient blood absorbability.^[^ ^35^ ^]^ Herein, we developed an AgNW‐SF theranostic device with high blood absorption capacity and compared their hemostatic efficacy with a commercial gelatin‐based hemostatic sponge, Dengen. To evaluate the hemostatic efficacy, the blood clotting experiment was conducted using whole human blood. Our results indicated that AgNW‐SF devices have a significant impact on blood clotting time. The time‐lapse picture of blood coagulation in **Figure** **6**A shows that all concentrations of the AgNW‐SF devices initiated blood clotting in just 2 min, which was significantly shorter than that for untreated blood (≈14 min), and in the order of clotting time for Dengen. The average blood clot formation time (i.e., hemostatic time) in Figure 6B presents a significant reduction in hemostatic time for all the experimental groups compared to the untreated blood control, with the best performance for AgNW‐SF (3%), followed by AgNW‐SF (4%) and AgNW‐SF (5%) devices, showing ≈87%, ≈82%, and ≈77% reduction in hemostatic time, respectively. Since the majority of commercial hemostats shorten the hemostatic time by ≈20–60%,^[^ ^36^ ^]^ the minimum 77% decrease in hemostatic time found by AgNW‐SF devices is a substantial and clinically significant hemostatic effect.

To quantify the hemostatic results, we measured the absorbance (at 542 nm) of the diluted supernatant collected from each sample after washing the excess blood (Figure 6C). As can be seen, the absorbance value for untreated blood was ≈4.5 at 3 min, and it was consistently maintained at the same level up to 7 min, indicating the absence of clot formation. Subsequently, at 14 min, the absorbance value dropped to ≈1.4, suggesting the formation of the blood clot. In contrast, the absorbance values for all concentrations of AgNW‐SF devices and Dengen were markedly lower (between 1 and 2) than that for untreated blood at 3 min, suggesting faster clot formation in the presence of the hemostat. By increasing the incubation time, the thickness of the blood clot increased, and the absorbance reading of the supernatant dropped and eventually reached the lowest level at 14 min. The quantification of absorbance values allowed us to more accurately measure and compare the degree of clot formation in each sample.

Furthermore, in vivo hemostatic efficacy of the AgNW‐SF devices was evaluated in a rat liver bleeding model. The 8 mm wound was created in the liver of rats using a biopsy punch, and AgNW‐SF devices were placed at the injury site to stop bleeding (Figure 6D and Movie S2, Supporting Information). Two experimental groups of hemostats, pure SF sponge and AgNW‐SF theranostic device at SF concentration of 4% w/v were selected for the in vivo test based on their strong mechanics, great blood absorbability, and bleeding detection sensitivity. Two control groups, a commercial hemostat, Dengen as a positive control, and an untreated injury model as a negative control were also included in the study. Any blood loss from the punctured lesion was collected on a filter paper beneath the liver for 2 min. As shown in Figure 6E, a smaller area of blood stain was observed on filter paper in the experimental group as compared with injury and Dengen control groups. To further quantify the amount of bleeding, the filter papers were weighed. As shown in Figure 6F, the total bleeding was ≈26% for pure SF sponge and ≈41% for AgNW‐SF devices, which were significantly lower than that for Dengen (≈76% bleeding). Moreover, the hemostatic time (blood coagulation time) was significantly reduced from ≈127 s for the injury group to ≈70 s for the experimental groups (Figure 6G). No significant changes were observed in the clotting time between the experimental groups and the commercial hemostat.

To further investigate the characteristics of the clots formed over the AgNW‐SF devices, we characterized the cellular compositions of the blood clot using SEM. At a lower magnification of the SEM image (Figure 6H‐i), a thick layer of the blood clot was visible, and the blood cells were penetrated into the porous structure of the device. At higher magnification (Figure 6H‐ii), fibrin filaments were visible, as indicated by arrows. These filaments were found to be a key component of the blood clot. In addition, we observed that activated platelets clumped together and became trapped under the fibrin network. SEM imaging further revealed that the clot components in the AgNW‐SF devices mainly consisted of RBCs, activated platelets, and a few white blood cells. These results provided further evidence for the hemostatic activity of the AgNW‐SF devices.

## 3 Conclusion and Outlook

Our AgNW‐SF device provides a previously unexplored strategy to construct an all‐in‐one theranostic platform by integrating the effective therapeutics of hemostasis and the diagnostics of the hemorrhage based on our in vitro and in vivo results. The device is biocompatible with a tunable in vivo biodegradation period that can be customized for long‐term use. Our results showed that the AgNW‐SF device is hemocompatible and can stop bleeding effectively, with a minor inflammatory response, in vivo. Also, the AgNW‐SF device itself can serve as a capacitive sensor for effective hemorrhage monitoring and could differentiate the whole blood from other bodily fluids (i.e., serum and water). In summary, our theranostic device could potentially address several challenges in hemorrhaging wounds: 1) Being biodegradable, cytocompatible, and hemocompatible; 2) Accelerating blood coagulation; 3) Preventing bacterial infection; 4) Detecting bleeding with a sandwich capacitive sensor; 5) Applicable for non‐compressible and irregular wounds; 6) Applicable for both topical and internal hemorrhage. Such a multifunctional device is highly promising for diagnosis and therapeutic applications in the clinic.

Future perspectives for this device include but are not limited to further optimization of the hydrophobic coating process to further improve the durability and stability of the AgNW electrodes. In addition, the device with a controllable lifetime and excellent manufacturability can be personalized,making it an attractive candidate for future in vivo studies to explore its full potential as a theranostic platform. Developing more advanced signal processing techniques (i.e., machine learning) and/or integrating other biosensors could enhance the device's specificity, accuracy, and practicability.

We envision that the AgNW‐SF theranostic device potentially upgrade the current clinical medical practice in the intelligent management of hemorrhage. The device's sensing capabilities could offer a timely alert on the early occurrence of bleeding. This could be particularly valuable when time is of the essence, enabling medical professionals to act quickly and effectively to stop bleeding and prevent possible mortality. This would also ensure that the patient is not at the risk for further hemorrhage or complications, especially after surgery. This theranostic platform could also be extended to other point‐of‐care applications by integrating specific biosensors (i.e., detecting other biomarkers of interest) and therapeutics (i.e., flexible drug delivery system).

## 4 Experimental Section

### Preparation of AgNW

According to a previous publication,^[^ ^16a^ ^]^ AgNW was produced by reducing silver nitrate. In short, a solution containing 1 g of polyvinylpyrrolidone (Sigma–Aldrich MO, USA) and 14 mg of sodium chloride (Sigma‐Aldrich MO, USA) in 20 mL of ethylene glycol (Sigma–Aldrich MO, USA) was stirred for 10 min and then heated to 160 °C for 20 min. A solution containing 10 mL of 0.14 m silver nitrate (Sigma–Aldrich MO, USA) was then slowly added to the mixture at a rate of 10 mL h^−1^ using a syringe pump. The reaction continued for 30 min at 160 °C, resulting in a greyish‐green mixture. To purify the mixture, it was washed twice with acetone and three times with Milli‐Q water. The resulting AgNW suspension was concentrated to 40 mg mL^−1^ (4% w/v) in pure ethanol (Fisher Scientific, PA, USA) and used in the experiment.

### Preparation of AgNW‐SF Device for Theranostic Applications

To fabricate AgNW‐SF devices, an aqueous suspension of commercial silk fibroin (Sigma‐Aldrich MO, USA) was prepared at concentrations of 3%, 4%, and 5% w/v and gently mixed by pipetting. An equal volume of the suspension (500 µL) was transferred to each well of a 48‐well plate. The plate was placed at −20 °C for 24 h to freeze, and the frozen samples were freeze‐dried (Labconco Medical Instrument Co., Ltd.) for 48 h. Subsequently, the samples were crystallized with CH_3_OH for 24 h at RT and rinsed with Milli‐Q water to remove the residual CH_3_OH. The samples were frozen and freeze‐dried for 48 h to create a porous SF sponge. The AgNW dispersion at a concentration of 40 mg mL^−1^ was prepared in ethanol and added to the filter assembly, and a vacuum pump was applied to transport AgNW to the filter. The SF sponges were placed on the filter, and the vacuum pump was used again to press the SF against the filter paper, resulting in coating the surface of the SF. The surface coating was also performed on the opposite side of the SF sponge to ensure that AgNW was deposited on both the top and bottom surfaces of the SF. The AgNW coating procedure was repeated three times until the desired electrical conductivity was attained. The resultant AgNW‐SF theragnostic device was stored in a moisture‐free environment at RT until use.

### SEM Imaging and Characterization

SEM was used to analyze the surface morphology and porous structure of the as‐synthesized and shape‐recovered AgNW‐SF devices. All the samples were freeze‐dried and sputter‐coated with a thin layer of gold using a sputter coater (Pelco SC‐7, USA) and examined under an SEM (Supra 40 VP, Zeiss, Germany). The microscope was operated at an accelerating voltage of 12 kV. The samples were imaged at a range of magnifications to capture the microstructure and surface features. The SEM images were analyzed using ImageJ software (Version 1.52e, USA) to quantify the pore size distribution. At least five different regions of each sample were imaged to ensure the representative morphology and size of the pores.

### FTIR Analyses

FTIR analyses of the SF sponge and AgNW‐SF theragnostic device were performed using attenuated total reflectance (ATR) mode. The freeze‐dried samples were placed on the diamond crystal surface of the FTIR spectrometer (Bruker ALPHA II, USA) for analysis. The FTIR spectra were collected within the range of 4000–400 cm^−1^ using 32 scans and a resolution of 4 cm^−1^. The background spectrum was collected before the analysis of each sample and subtracted from the spectra of the samples. The obtained spectra were analyzed using OPUS software (Version 8.7.31, Bruker, USA). The functional groups and chemical bonds in each sample were identified by comparing the obtained spectra with standard FTIR spectra from the database.

### Mechanical Test

Mechanical strength of the AgNW‐SF theranostic devices at varying concentrations (3%, 4%, and 5% w/v) of SF was assessed using a universal mechanical testing machine (Model 5943, MA, USA). Cylindrical shape devices with a diameter of 10 mm and height of 8 mm were prepared and soaked in Milli‐Q water 15 min prior to the experiments. Compression testing at the rate of 2 mm min^−1^ was performed to obtain stress‐strain curves using Bluehill software (Version 3, USA). The slope of the first 10% of the stress–strain curve was calculated as compressive modulus. To investigate the cyclic compression properties of the AgNW‐SF theranostic devices, the hydrated devices were subjected to a 50‐cycle compressive load at a speed of 5 mm min^−1^ and strain of 50%. The force‐displacement curve and stress–time graph were plotted.

### Shape‐Recovery and Fluid Absorption Test

Cylindrical shape AgNW‐SF devices with a diameter of 10 mm and height of 8 mm were prepared and soaked in Milli‐Q water or blood to saturate completely. Subsequently, the devices were compressed to squeeze out the water/blood. The extra water/blood was blotted to achieve a shape‐fixed compressed structure. Next, freshwater or blood was added to the compressed samples until they recovered. The positions of the samples at each step were captured by a digital camera. To quantify the amount of water absorbed by the device, the dry weight of the devices was recorded (*W*_d_). The devices were then immersed in water, and their weight was measured at various time intervals, referred to as the wet weight (*W*_w_). The water absorption (%) was calculated by [(*W*_w_−*W*_d_)/*W*_d_]×100. The dimensions of the device before and after water absorption were measured, and the volume of the device was calculated to investigate if any swelling occurred. The slope of the water or blood absorption curve within the first 1 min was calculated to determine the water/blood absorption rate.^[^ ^5a^ ^]^

### Hemocompatibility Assessment

To evaluate the hemocompatibility of the AgNW‐SF devices, a hemolysis assay was performed following a previously published protocol to observe and measure hemoglobin release.^[^ ^28a^ ^]^ Citrated human whole blood was purchased from Zenbio, NC, USA. Fiftyfold diluted blood was prepared using a 0.9% w/v saline solution (Teknova, CA, USA). Samples were placed in Eppendorf tubes, and 2 mL of the diluted blood was added to each sample, followed by incubation at 37 °C for 2 h. The tubes were then centrifuged at 14 000 rpm for 10 min, and the resulting supernatants (100 µL of each sample) were transferred into a 96‐well plate. The absorbance was measured at 542 nm using a microplate reader (BioTeK, UV/Vis Synergy 2, VT, USA). Negative and positive controls were established using saline and Triton X‐100 (1% v/v), respectively. Hemolysis percentage was calculated as *H*% = [(*A*_sample_ − *A*_negative_)/*A*_positive_]×100, where *A*_sample_ is the absorbance of the supernatant containing AgNW‐SF, A_negtive_ is the absorbance of the saline‐diluted blood, and *A*_positive_ is the absorbance of the Triton X‐100 containing blood. The hemolysis assay was conducted twice in triplicates. The mean hemolysis ratio (%) ± standard deviation of all replicates is reported.

### Cytocompatibility Test

HDF cells (ATCC, VA, USA) were cultured in Dulbecco's modified eagle medium (Gibco, NY, USA), supplemented with 10% v/v fetal bovine serum (Gibco, NY, USA), and 1% v/v streptomycin‐penicillin (Gibco, NY, USA). The cells were grown in a 5% CO_2_ incubator (Fisher Scientific, PA, USA) at 37 °C until 90% confluency, and then 10^4^ cells were seeded on the surface of the sponge/device in a 24‐well tissue culture plate. The cells which were directly seeded in the wells served as control.

PrestoBlue Cell Viability Reagent (Fisher Scientific, PA, USA) was used to assess the cytotoxicity of pure SF sponges and AgNW‐SF devices on days 1, 3, and 5. Briefly, PrestoBlue reagent was diluted at the ratio of 1:9 with cell medium, added to each sample (1.5 mL), and incubated for 1.5 h at 37 °C in the dark. Later, 100 µL of the supernatant from each sample was transferred to a 96‐well plate. The fluorescence intensity was measured using a microplate reader at an excitation of 530 nm and an emission of 590 nm. The results are reported after subtracting the background signal (i.e., PrestoBlue reagent without cells). The fluorescence intensity of the samples was normalized to the controls to calculate cell viability. The data are presented for 4 replicates ± standard deviation.

On day 5 of incubation, the samples were washed with Dulbecco's phosphate‐buffered saline (DPBS, Gibco, NY, USA), and Live/Dead viability/cytotoxicity assay (Invitrogen, supplied by Fisher Scientific, PA, USA) was conducted. The live/dead staining solution was prepared by mixing ethidium homodimer‐1 (20 µL) and calcein AM (5 µL) in DPBS (10 mL). The staining solution was added to each sample (500 µL) and incubated for 15 min in the dark at 37 °C. After washing the samples with DPBS, fluorescence microscopy (Keyence, BZ‐X700 Series) images were captured using the red channel for ethidium homodimer‐1 and the green channel for calcein AM.

### In Vitro Anti‐Infective Assay

The in vitro anti‐infective efficacy against *S. aureus* (ATCC, 25923) and *E. coli* (ATCC, 25922) was investigated using a zone inhibition assay, as reported earlier.^[^ ^27^, ^37^ ^]^ The experimental materials, including a 4% SF sponge and AgNW‐SF (4%), were prepared (dimension of 10 mm and thickness of 2 mm), soaked in Milli‐Q water for 1 week, and subsequently freeze‐dried. The freeze‐dried samples were sterilized under UV irradiation (wavelength = 250 nm) for 1 h prior to the experiment. Next, the optical density (OD) of the bacterial inoculum was read at 600 nm using Nanodrop (Fisher Scientific PA, USA) and adjusted to 0.2 by diluting in Bacto tryptic soy broth (Fisher Scientific PA, USA). The diluted bacterial inoculum (150 µL) was evenly spread on a sterile tryptic soy agar (Fisher Scientific PA, USA) plate using a cell spreader. The sterilized sponges/devices were placed on the agar plate. To serve as a positive control, a filter paper containing 60 µg of tetracycline solution was also included in each agar plate. The plates were then incubated at 37 °C for 12 h. The clear zone around each sample was measured, and the area of inhibition was calculated and compared with the positive control to assess the percentage of growth inhibition.

Further, a bacterial LIVE/DEAD viability/cytotoxicity kit (Fisher Scientific, PA, USA) was used to fluorescently label live and dead bacteria. For this purpose, 1 mL of bacterial inoculum (OD_600_ = 0.2) was added to each sponge/device in the 24‐well plate and incubated for ≈6 h at 37 °C. After incubation, the supernatant from each well was collected and transferred to an Eppendorf tube. The tube was then centrifuged at 12000 rpm for 1 min at RT, and the resulting pellets were washed with DPBS and resuspended in 500 µL DPBS. Live/dead staining was performed by adding SYTO 9 (green fluorescence) and propidium iodide (red fluorescence) to the bacteria suspension at a concentration of 3 and 15 µm, respectively. The mixture was incubated in the dark for 15 min at 37 °C. Next, 5 µL of the mixture was placed on a glass slide and covered with a cover glass. The sample was then imaged using a Keyence fluorescence microscope under green and red channels.

### In Vitro Blood Clotting Test

To assess the clotting ability of AgNW‐SF, sodium citrate anticoagulated human blood (Zenbio NC, USA) was obtained and used in the experiment within 48 h after withdrawal. The clotting test was carried out in a 48‐well plate by mixing 0.1 m calcium chloride (Teknova, CA, USA) with the whole blood at a 1:9 ratio, shaking for 10 s, and pipetting 250 µL onto each AgNW‐SF sample, and Dengen, commercial gelatin‐based hemostatic sponge. The blood added to the sample‐free wells was used as a control. After adding blood, the wells were gently washed with normal saline at predetermined time intervals (0–14 min) until whole soluble blood was cleaned away and only the clot remained in the wells. Clotting time was determined as the time at which a uniform clot was formed in the samples/wells, excluding the 10 s of shaking. The clotting time for AgNW‐SF or Dengen samples was then compared to that of untreated blood. At 3, 4, 5, 6, 7, and 14 min of clotting time, 10 mL of saline was added to the clot, and the absorbance was read at 542 nm using a microplate reader to determine the degree of clot formation. The lower absorbance value corresponds to the higher degree of clot formation.

### In Vivo Systemic Toxicity Evaluation

The animal trials conducted in this study were approved by the Lundquist Institute Animal Care and Use Committee (#22590/91‐02) and were carried out in accordance with authorized protocol.^[^ ^38^ ^]^ Male Sprague‐Dawley rats that were seven weeks old and weighed between 250 and 300 g were obtained from Charles River Laboratories (CA, USA) and were housed in a certified animal facility. The rats were anesthetized with 1.5% isoflurane (Piramal, PA, USA) in oxygen for the biocompatibility evaluation study, and their dorsal sides were shaved and cleaned with 0.2% w/v iodophor (Fisher Scientific, PA, USA) and 70% v/v ethanol to avoid hair contamination. Two subcutaneous pockets were created on either side of the longitudinal incision (2 cm) made on the posterior dorsal skin. One sponge or AgNW‐SF device (10 mm diameter, 4 mm thickness) was implanted in each pocket (n = 4 per animal). Prior to implantation, all the samples were UV sterilized (wavelength of 254 nm, duration 1 h). The incision was closed with surgical staples/clips after implantation. The experimental groups consisted of two groups: 1) AgNW‐SF (4%) and 2) Pure 4% SF sponge. Two control groups were also included: 1) Untreated injury control with no implants and 2) Dengen commercial hemostatic sponge (positive control). After 2 and 4 weeks of implantation, the animals were euthanized by CO_2_ inhalation to examine the systemic toxicological reaction to the main organs. Vital organs such as the liver, heart, lung, spleen, and kidney were collected immediately after sacrificing the animals and placed in 10% v/v neutral buffered formalin (Leica Biosystems, IL, USA). Fixed organs were cut into 8 µm thick sections, treated using standard methods, and embedded in paraffin. Histology images were taken using a Keyence (BZ‐X710, IL, USA) inverted microscope after staining paraffin‐embedded tissues with H&E.

### Immunofluorescence Staining

The tissue sections were deparaffinized and rehydrated in a series of ethanol (50–100% v/v). The heat‐induced epitope retrieval method was employed for 30 min using a citrate‐buffered antigen retrieval buffer (1×). The tissues were permeabilized with TBST (tris‐buffered saline + 0.25% Triton‐X‐100, Abcam, UK) and blocked with 10% w/v bovine serum albumin (Sigma–Aldrich MO, USA) for 2 h at RT. The slides were then incubated with primary antibodies, including anti‐CD3 rabbit monoclonal antibody (Abcam, ab1669) at a dilution of 1:200 in TBST and anti‐CD68 Alexa fluor‐488 labeled mouse anti‐rat monoclonal antibody (PIMA528262) at a dilution of 1:500 in TBST, for 4 h at RT. A secondary antibody to anti‐rabbit Alexa 594 (Abcam, ab150080) was used at a dilution of 1:500 in TBST. Nuclei were visualized by mounting sections with an antifade mounting solution containing DAPI (Vector Laboratories, USA). Images were captured using the Echo Revolution microscope.

### Masson's Trichrome staining

The Masson's Trichrome staining was performed following the manufacturer's protocol (Electron Microscopy Sciences, Catalog #: 26367‐Series). Briefly, the paraffinized sections were deparaffinized and hydrated with distilled water. The hydrated sections were fixed with 4% paraformaldehyde (Santa Cruz Biotechnology, USA) at RT for 1 h and rinsed with distilled water. The sections were stained with Weigert's iron hematoxylin working solution for 5 min and washed with running water. Later, they were placed in Biebrich Scarlet‐Acid Fuchsin (#26033‐25) and Phosphomolybdic Acid‐Phosphotungstic Acid for 15 min each and rinsed with distilled water. Next, the sections were stained in Aniline Blue Solution (#26367‐06) for 20 min and then differentiated in 1% Acetic Acid (#26367‐07) for 5 min. The stained slides were dehydrated and mounted. The mosaic stitched images were captured using Echo Revolution bright field microscope to visualize the entire scaffold at 20× magnification.

### In Vivo Liver Bleeding Model

To evaluate the hemostatic function of AgNW‐SF devices, the rat liver injury model was used following previously published protocols.^[^ ^28^, ^39^ ^]^ Twenty male Sprague–Dawley rats, aged 7 weeks, were used to conduct in vivo bleeding models. The ventral side of the hair was clipped and disinfected under inhalation anesthesia. A 2 cm incision was made below the ribcage to expose the liver outside the body. Excess blood was absorbed using sterilized gauze, and a filter paper was weighed and placed around the exposed liver lobe. A biopsy punch (8 mm) was used to puncture the liver, and the sponges (10 mm diameter, 8 mm thickness) were placed on the bleeding site. Once it was confirmed that the bleeding had stopped at the lesion site (2 min), the weight of the filter paper that had absorbed blood was measured. The amount of bleeding was determined by subtracting the weight of the wet filter paper from the weight of the dry paper. The bleeding percentage was calculated as [(Weight of blood loss in experimental animal)/Weight of blood loss in injury control]×100. The control groups included a negative control group (punctured liver without treatment or injury) and a positive control group treated with a commercialized hemostatic sponge (Dengen). Two experimental groups consisting of pure 4% SF sponges and AgNW‐SF (4%) theranostic devices were used to evaluate the hemostatic efficacy as compared with the control. The minimum time required to form a blood clot in each group was monitored with a stopwatch. The data are reported as mean values of 5 replicates in each group ± standard deviation.

### Sheet Resistance and Capacitance Measurement

To measure resistance of the devices independent of the AgNW coating thickness, sheet resistance was calculated. For this reason, the two electrodes of the commercial Fluke multimeter were horizontally placed on the surface of AgNW‐SF devices in such a way that 7 mm length of each commercial electrode was in contact with the material. The distance between two commercial electrodes on the surface of the material was 4 mm. The resistance was measured in Ohm (Ω). Accordingly, the sheet resistance (Ω sq^−1^) was calculated by (Resistance × Contact length of the electrode)/Distance between two electrodes. The capacitance was measured using Potentiostat/Galvanostat/ZRA (Gamry instruments, PA, USA) at a frequency of 10 Hz and AC voltage of 100 mV RMS.

### Statistical Analyses

Statistical analysis for all tests was carried out using GraphPad Prism software (Version 9, USA), with at least three samples in each group for each experiment. The results are presented as mean values ± standard deviation. The unpaired two‐tailed t‐test was used to compare two groups, while one‐way ANOVA with Tukey's multiple comparison tests or two‐way ANOVA were used for multiple group comparison, when appropriate. The significance level was denoted by an asterisk in the figure caption, wherever applicable.

## Conflict of Interest

The authors declare no conflict of interest.

## Author Contributions

R.H. and Y.Z. came up with the original idea and designed the experiments. R.H. and A.G. contributed equally to this work and conducted and analyzed most of the experiments, and prepared the original draft with assistance from Y.Z. H.M. and Z.L. helped with sensor verification and mechanical test analyses. F.Z. contributed to the biodegradation test and partial writing of the experimental section. M.E. helped in cytocompatibility experiments and related data analyses. N.R.B. and K.M. helped to draw schematics. Y.D., F.Z., and S.K. assisted in animal tissue sectioning and antibacterial experiment. J.L. contributed to the device preparation. H.‐J.K. guided for in vivo study and data analyses. The study was conceived of and supervised by R.H., A.K., and Y.Z.

## Supporting information

Supporting Information

Supplemental Movie 1

Supplemental Movie 2

## Data Availability

The data that support the findings of this study are available from the corresponding author upon reasonable request.
