# Supplementary material for: An All‐In‐One Transient Theranostic Platform for Intelligent Management of Hemorrhage
Source: Adv Sci (Weinh). 2023 Jun 4;10(24):2301406. doi: 10.1002/advs.202301406 (PMC10460878; doi:10.1002/advs.202301406)
Supplement: Supplementary file 1 — Supporting Information [file ADVS-10-2301406-s002.pdf]

ADVANCED SCIENCE
Open Access

Supporting Information

for *Adv. Sci.*, DOI 10.1002/advs.202301406

An All-In-One Transient Theranostic Platform for Intelligent Management of Hemorrhage

Reihaneh Haghniaz, Ankit Gangrade, Hossein Montazerian, Fahimeh Zarei, Menekse Ermis, Zijie Li, Yuxuan Du, Safoora Khosravi, Natan Roberto de Barros, Kalpana Mandal, Ahmad Rashad, Fatemeh Zehtabi, Jinghang Li, Mehmet R. Dokmeci, Han-Jun Kim, Ali Khademhosseini* and Yangzhi Zhu**

Supporting Information

An All-In-One Transient Theranostic Platform for Intelligent Management of Hemorrhage

*Reihaneh Haghniaz,*Ankit Gangrade, Hossein Montazerian, Fahimeh Zarei, Menekse Ermis Sen, Zijie Li, Yuxuan Du, Safoora Khosravi, Natan Roberto de Barros, Kalpana Mandal, Ahmad Rashad, Fatemeh Zehtabi, Jinghang Li, Mehmet R. Dokmeci, Han-Jun Kim, Ali Khademhosseini*, Yangzhi Zhu**

R. Haghniaz, A. Gangrade, H. Montazerian, F. Zarei, M. Ermis Sen, Z. Li, Y. Du, S. Khosravi, N. R. Barros, K. Mandal, A. Rashad, F. Zehtabi, J. Li, M. R. Dokmeci, H-J. Kim, Y. Zhu, A. Khademhosseini

Terasaki Institute for Biomedical Innovation, Los Angeles, CA 90064, USA

E-mail: rhaghniaz@terasaki.org; khademh@terasaki.org; yzhu@terasaki.org;

H. Montazerian

Department of Bioengineering, University of California, Los Angeles, CA 90095, USA

California NanoSystems Institute, University of California, Los Angeles, CA 90095, USA

Z. Li, Y. Du

Mork Family Department of Chemical Engineering & Materials Science Viterbi School of Engineering University of Southern California, Los Angeles, CA 90007, USA

S. Khosravi

Electrical and Computer Engineering Department, University of British Columbia, Vancouver, BC V6T 1Z4, Canada

H-J Kim

College of Pharmacy, Korea University, Sejong, 30019, Republic of Korea.

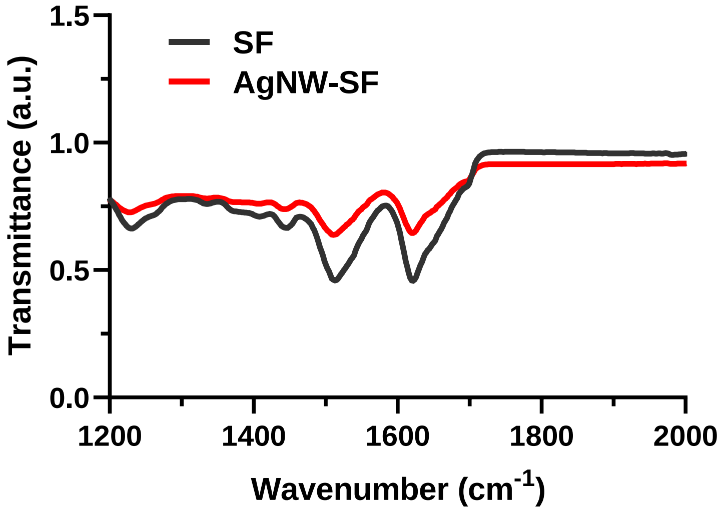

Figure S1. Fourier transform infrared spectroscopy (FTIR) spectra of SF sponge and AgNW-SF theranostic device.

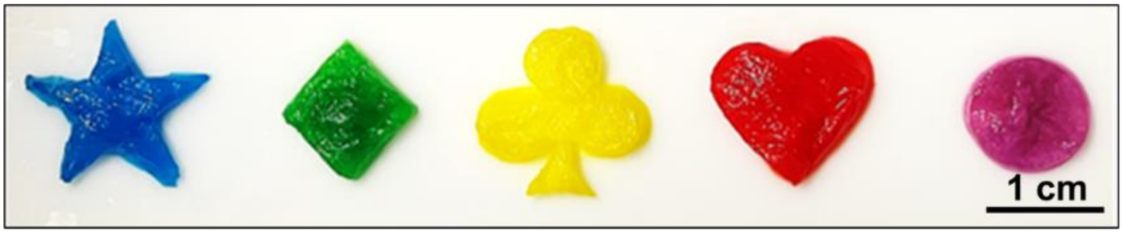

Figure S2. Manufacturability of the AgNW-SF theranostic device by laser engraving.

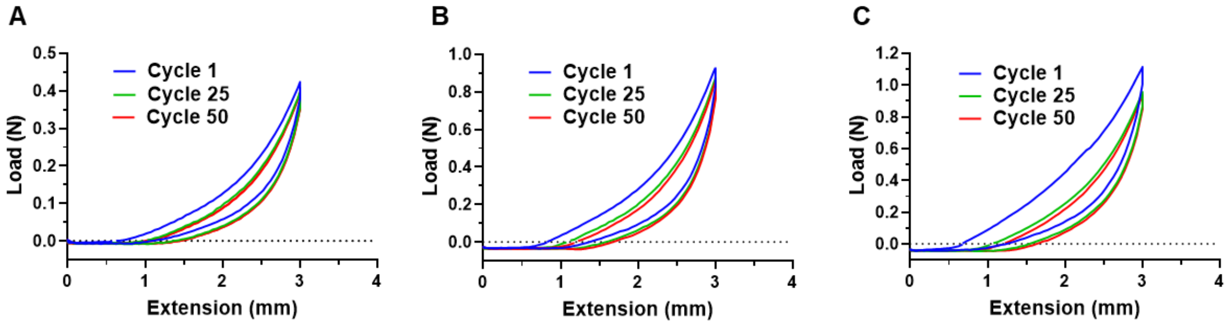

Figure S3. The cyclic compression load-extension curves for hydrated (A) AgNW-SF (3%), (B) AgNW-SF (4%), and (C) AgNW-SF (5%).

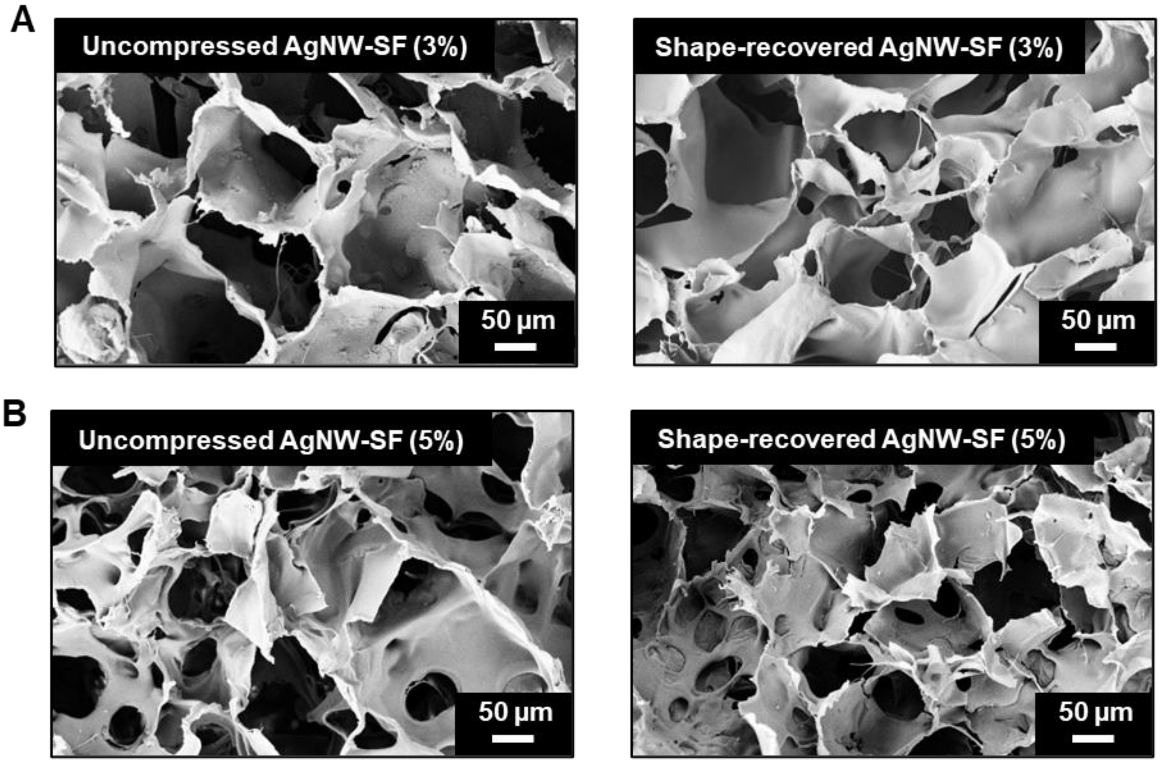

Figure S4. Scanning electron microscopy (SEM) images of the AgNW-SF theranostic devices at (A) 3% SF concentration, and (B) 4% SF concentration under compression and shape recovery.

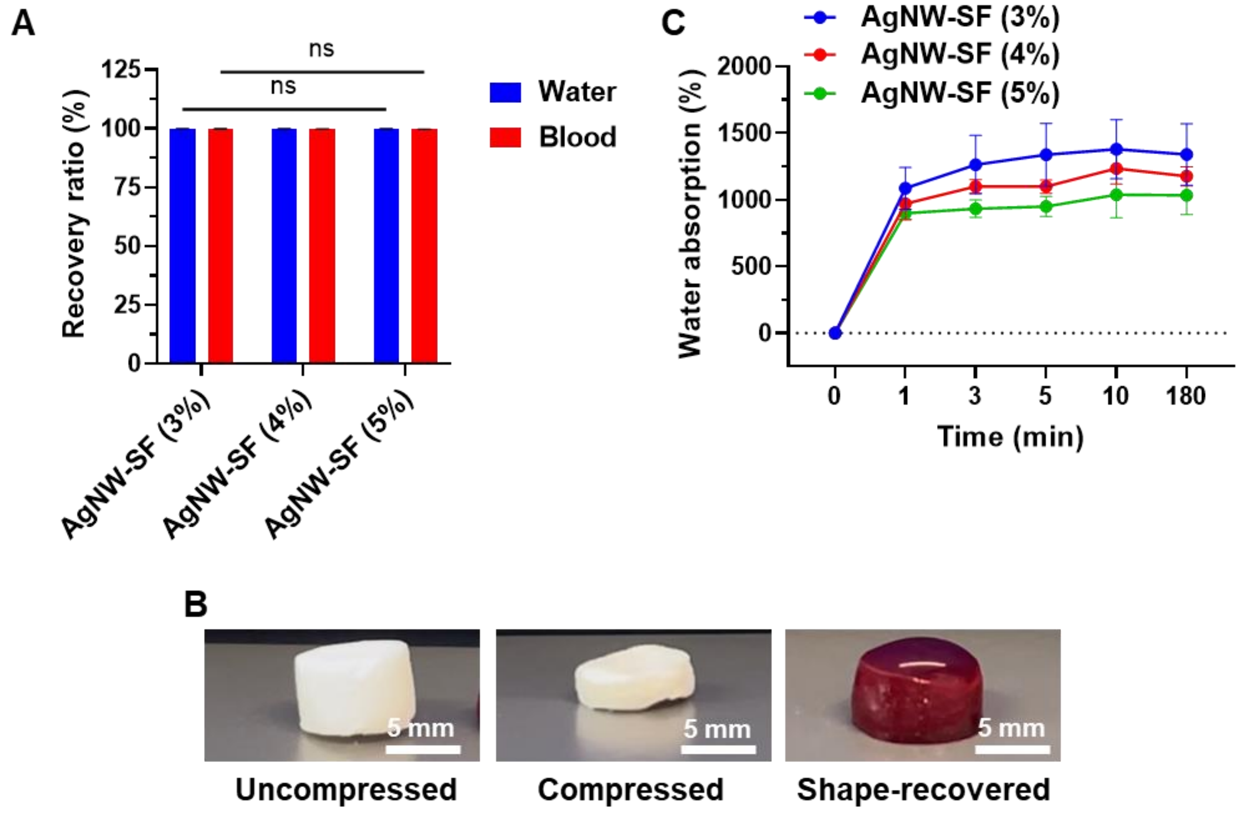

Figure S5. Shape-recovery property of the AgNW-SF theranostic devices. (A) Shape recovery ratio of the compressed AgNW-SF devices after absorption of water or blood. (B) Photograph of the blood-triggered shape-recovered AgNW-SF (4%) after freeze-drying the compressed structure. (C) Water absorption rate of the AgNW-SF devices at different concentrations of SF. The significant differences were analyzed for $n=4$, using one-way ANOVA, followed by Tukey's multiple comparisons test. Asterisks indicate statistically significant results with p-values <0.05 (*), <0.01 (**), <0.001 (***), or <0.0001 (****). The 'ns' indicates no significant differences.

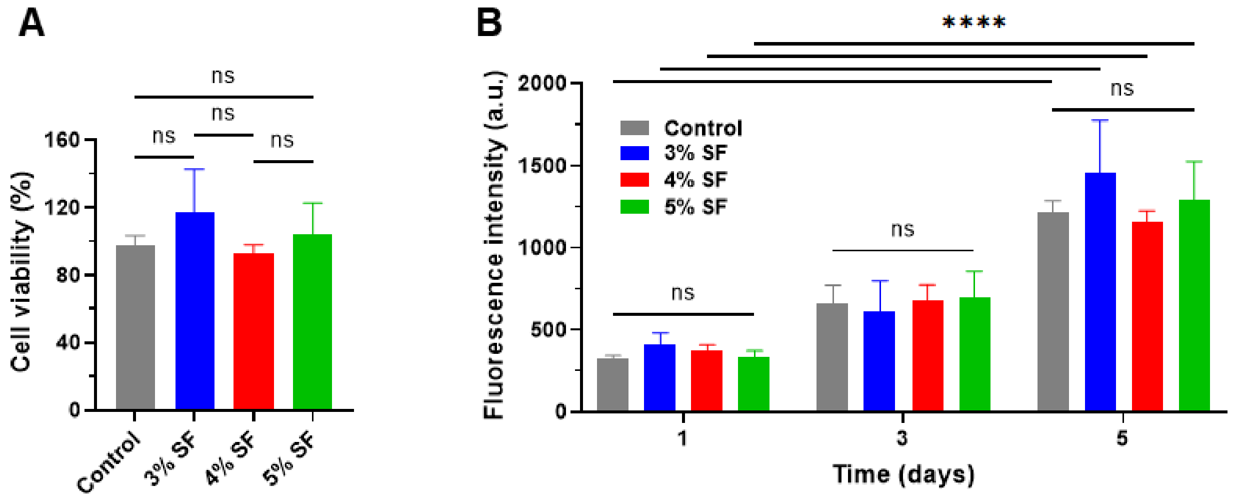

Figure S6. (A) Quantified human dermal fibroblast (HDF) cell viability on day 5 of incubation with varying concentrations of pure silk fibroin (SF) sponges. (B) The fluorescence intensity obtained by PrestoBlue^{TM} assay after 1, 3, and 5 days of incubation of the HDF cells with varying concentrations of SF. Data are shown for 4 replicates ± standard deviation. When appropriate, the significant differences were analyzed by one-way, or two-way ANOVA followed by a multiple comparisons test. Asterisks indicate statistically significant results with p-values <0.05 (*), <0.01 (**), <0.001 (***), or <0.0001 (****). The 'ns' indicates no significant differences.

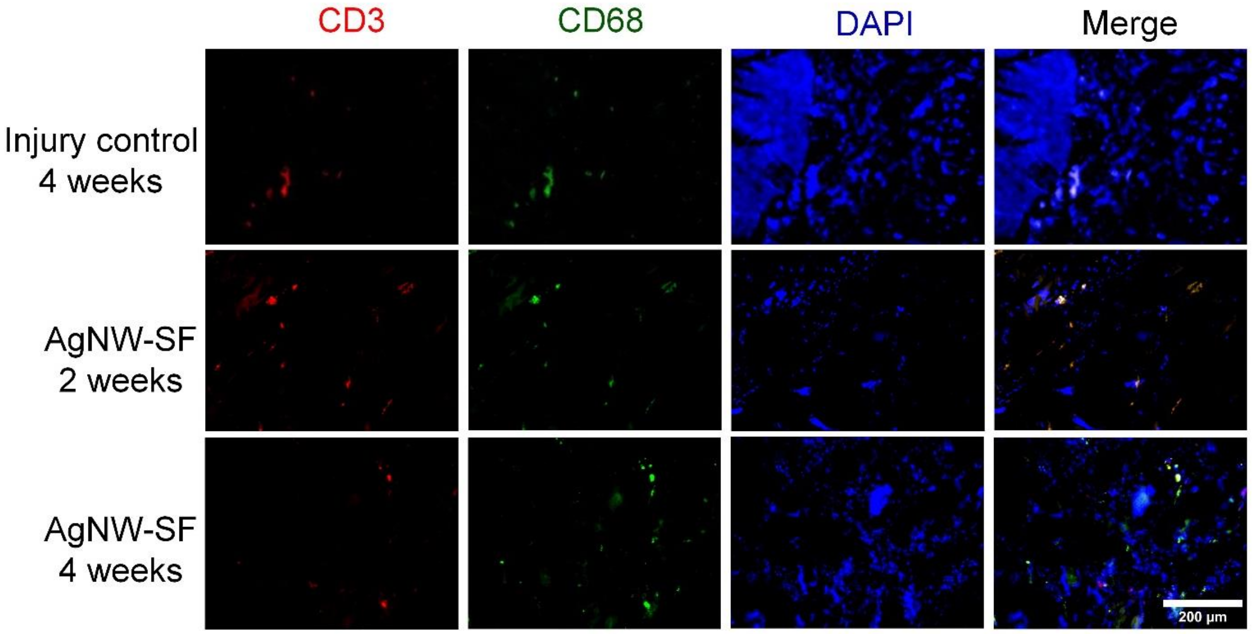

Figure S7. Immunofluorescence staining of inflammatory cells in implantation experiment. The fluorescence images show CD3 (T cells, in red), CD68 (macrophages, in green), and DAPI (nuclei, in blue) in the experimental groups 2 weeks and 4 weeks after subcutaneous implantation of AgNW-SF devices.

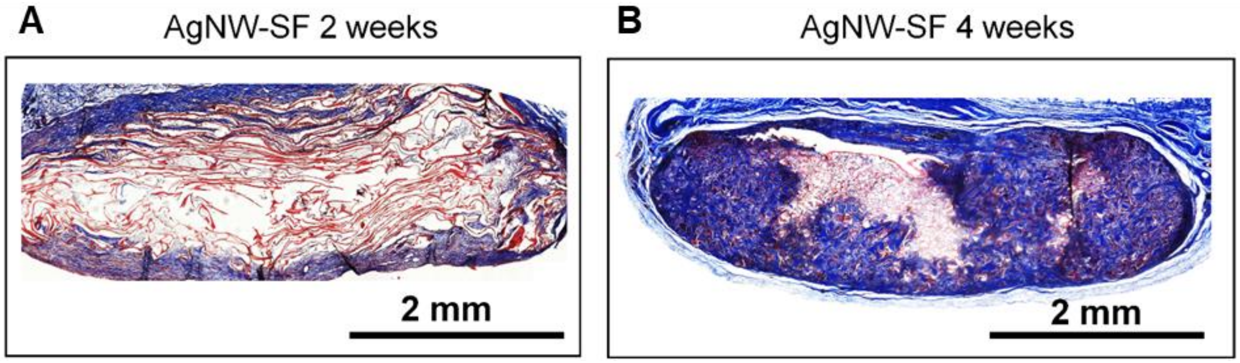

Figure S8. The Masson's Trichrome staining of extracellular matrix. Cross-section images of the AgNW-SF devices stained with Masson’s Trichrome, A) 2 weeks after implantation and B) 4 weeks after implantation. Blue stains show the deposition of collagen inside the spongy device.

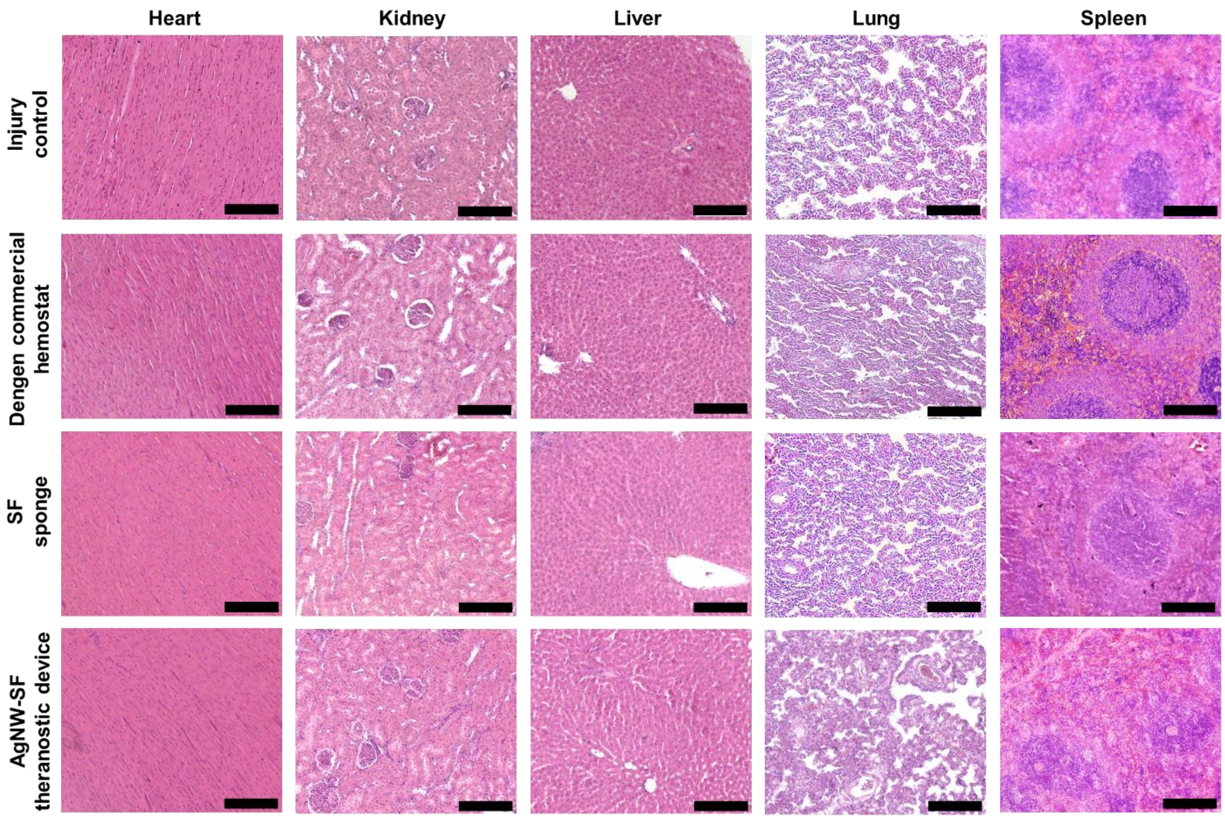

Figure S9. *In vivo* systemic toxicity assessment of AgNW-SF theranostic devices. Images of the main tissues (Heart, kidney, liver, lung, spleen) stained with hematoxylin and eosin (H&E) 2 weeks after subcutaneous implantation of Dengen® (commercial hemostat), pure SF sponge or AgNW-SF theranostic devices in rats. Scale bars show 200 μm.

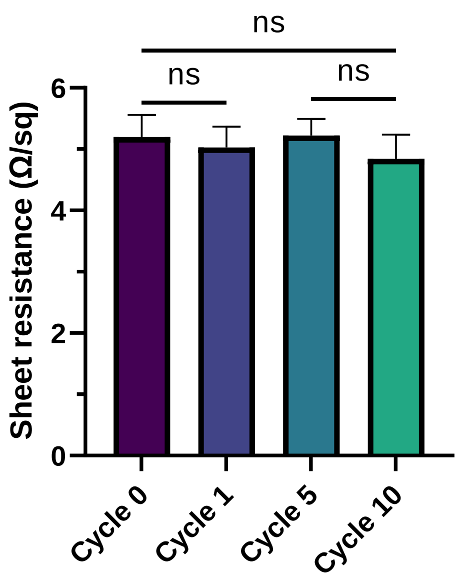

Figure S10. Sheet resistance of the AgNW-SF device (4%) which are measured after different cycles of hydration in Milli-Q water and subsequent freeze-drying. Data are shown for 3 measurements. The significant differences were analyzed by one-way ANOVA, followed by Tukey's multiple comparisons test. The 'ns' indicates no significant differences.

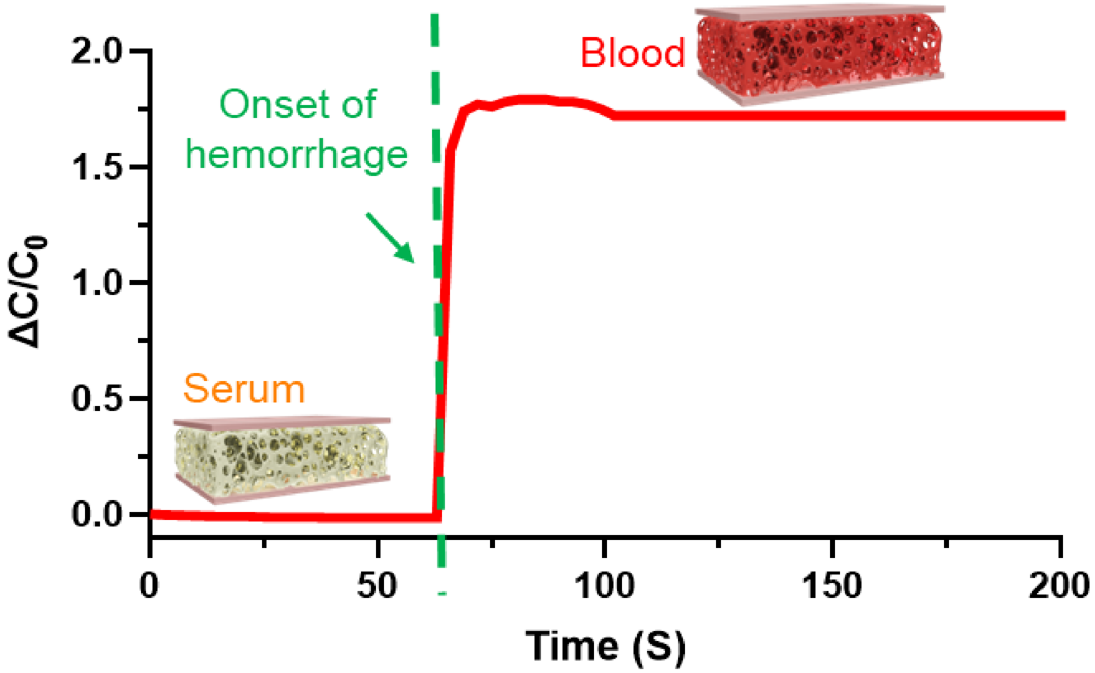

Figure S11. *In vitro* monitoring of the hemorrhage with AgNW-SF theranostic device. Continuous monitoring of hemorrhage by distinct variation in capacitance after absorption of blood by the device saturated in serum.
